# Influence of lean and fat mass on bone mineral density and on urinary stone risk factors in healthy women

**DOI:** 10.1186/1479-5876-11-248

**Published:** 2013-10-07

**Authors:** Antonio Nouvenne, Andrea Ticinesi, Angela Guerra, Giuseppina Folesani, Franca Allegri, Silvana Pinelli, Paolo Baroni, Mario Pedrazzoni, Giuseppe Lippi, Annalisa Terranegra, Elena Dogliotti, Laura Soldati, Loris Borghi, Tiziana Meschi

**Affiliations:** 1 Department of Clinical and Experimental Medicine, University of Parma, Via A. Gramsci 14, Parma 43126, Italy; 2 Department of Mathematics, Uppsala University, Uppsala, Sweden; 3 Laboratory of Clinical Chemistry and Haematology, Department of Pathology and Laboratory Medicine, Parma University Hospital, Parma, Italy; 4 Department of Health Sciences, Università degli Studi di Milano, Milan, Italy

**Keywords:** Body composition, Lean mass, Fat mass, Bone mineral density, Urinary lithogenic risk factors

## Abstract

**Background:**

The role of body composition (lean mass and fat mass) on urine chemistries and bone quality is still debated. Our aim was therefore to determine the effect of lean mass and fat mass on urine composition and bone mineral density (BMD) in a cohort of healthy females.

**Materials and methods:**

78 female volunteers (mean age 46 ± 6 years) were enrolled at the Stone Clinic of Parma University Hospital and subdued to 24-hour urine collection for lithogenic risk profile, DEXA, and 3-day dietary diary. We defined two mathematical indexes derived from body composition measurement (index of lean mass-ILM, and index of fat mass-IFM) and the cohort was split using the median value of each index, obtaining groups differing only for lean or fat mass. We then analyzed differences in urine composition, dietary intakes and BMD.

**Results:**

The women with high values of ILM had significantly higher excretion of creatinine (991 ± 194 vs 1138 ± 191 mg/day, p = 0.001), potassium (47 ± 13 vs 60 ± 18 mEq/day, p < 0.001), phosphorus (520 ± 174 vs 665 ± 186 mg/day, p < 0.001), magnesium (66 ± 20 vs 85 ± 26 mg/day, p < 0.001), citrate (620 ± 178 vs 807 ± 323 mg/day, p = 0.002) and oxalate (21 ± 7 vs 27 ± 11 mg/day, p = 0.015) and a significantly better BMD values in limbs than other women with low values of ILM. The women with high values of IFM had similar urine composition to other women with low values of IFM, but significantly better BMD in axial sites. No differences in dietary habits were found in both analyses.

**Conclusions:**

Lean mass seems to significantly influence urine composition both in terms of lithogenesis promoters and inhibitors, while fat mass does not. Lean mass influences bone quality only in limb skeleton, while fat mass influences bone quality only in axial sites.

## Introduction

The role of body weight, body mass index and body composition in the evaluation of lithogenic risk is still controversial. Even if many studies show an increase in the risk of developing nephrolithiasis with higher levels of BMI, the exact contribution of lean mass and fat mass in determining this risk is still unclear.

There are some large epidemiologic studies recording a rise in the risk of kidney stones as body weight, BMI and abdominal circumference increase [1,2]. The rise in the risk is however associated to a change in the type of nephrolithiasis, with the prevalence of calcium stones decreasing and the prevalence of uric acid stones increasing [3-5]. A rise in lean body mass has been linked to an increase in the incidence of nephrolithiasis only in male [6]. Moreover, a loss of weight is not associated to a decline in the risk [2].

On the other hand, if we consider urinary factors of lithogenic risk, an inverse correlation between pH and BMI and between pH and fat mass has been reported [7]. Moreover, the excretion of oxalate has been linked to body weight, body surface area and to lean mass [8]. The excretion of oxalate, uric acid, sodium, phosphate and calcium rises when BMI increases [9-11]; however, the calcium excretion loses significance after correction for sodium and phosphate [9].

Some recent studies have also shown a positive relation between urinary lithogenic risk factors, overweight and obesity [12-14]. Nevertheless, relative supersaturations are not altered since even inhibitor excretion and water intake increase with the body weight and/or BMI percentiles growth. A common limit of many of these studies is the lack of a precise evaluation of dietary habits, particularly in protein intake [15,16].

Even the relation between body composition and bone mineral density is debated. It has been demonstrated that an increase in body weight improves bone mineral density, but the specific role of lean mass and fat mass remains uncertain such as different effects in men and women [17]. There is a positive relation, proven in many studies, between fat mass and vertebral bone mineral density, while lean mass seems to be related to a higher bone mineral density only in some areas and is highly influenced by age and physical exercise [18-20]. On the other hand a link between urine chemistries, body composition and bone mineral density has been described [21,22].

In this paper, basing on a cohort of healthy women, we identified two new mathematical indexes pointing out the role of lean body mass and fat body mass separately, trying to eliminate possible confounding factors (e.g. height) present in already validated indices such as the Fat-Free Mass Index and Fat Mass Index [23]. Therefore we verified: 1) whether the urinary excretion of lithogenic risk factors is influenced by the whole body weight or by its composition in lean mass and fat mass; 2) how the bone mineral density is related to body composition; 3) which are the body areas where lean mass or fat mass most influence bone mineral density.

## Materials and methods

### Participants

We studied 78 healthy female volunteers at the Stone Clinic of the Clinical and Experimental Medicine Department, Parma University Hospital, Italy. Approvation by Ethical Committee of Parma Province was obtained as well as written informed consent from the patient for the publication of this report and any accompanying images. The study was carried out in compliance with the Helsinki Declaration.

All women carried out: 1) 24-hour urine collection for the laboratory determination of urinary lithogenic risk factors; 2) bone mineral density and body composition measurement through Dual-Energy X-Ray Absorptiometry with a fan-beam Hologic QDR 4500A densitometer (Hologic, Bedford, Mass., USA); 3) 3-day dietary diary in non-consecutive days with one corresponding to the day of the urinary collection, subsequently analyzed by a dietitian and interpreted with a specific software (Dietosystem, DS Medica, Milano, Italy).

### Densitometry

Body Composition was measured by DEXA with a fan beam densitometer (Hologic QDR 4500 A) and dedicated software (rel. 8.2). In the DEXA measurement, a trained physician performed the measurements on the women. DEXA measurements were performed following standard procedures, according to the manufacturer’s guidelines, while the participant was lying in a supine position. The trunk was considered as the region delimited by a horizontal line passing under the chin, two vertical lines passing through the medial margin of the head of the homerus, excluding all of the upper limbs, and two oblique lines at the groin cutting midway through the neck of the femur and crossing below the pubis. Intra-site repeatability was a mean of 2–3 & for FM. The coefficients of variation for the method were assessed by repeated measurements.

### Definition of mathematical indexes for body lean mass and body fat mass

#### Index of lean mass (ILM)

We defined an index in order to obtain two groups of women not different for body weight and BMI but only for lean mass. Body weight (BW), lean mass (LM) and fat mass (FM) are not independent variables because we can assume that total body weight is the sum of lean mass and fat mass.

ILM has been conceived for not being influenced by body weight, i.e.:

Since

BW=LM+FM

Then

$$\mathrm{ILM}=\mathrm{BW}\times \left(\mathrm{LM}–\mathrm{FM}\right)$$

That we can also write as

$$\mathrm{ILM}=\left(\mathrm{LM}+\mathrm{FM}\right)\times \left(\mathrm{LM}–\mathrm{FM}\right)$$

And therefore

$$\mathrm{ILM}={\mathrm{LM}}^{2}–{\mathrm{FM}}^{2}$$

Since every woman studied has a lean body mass higher than the fat body mass, elevating their values to the power of two we obtain the higher difference the heavier the lean mass is.

Therefore we calculated the value of ILM for every woman involved in the study, we found its median and we split our population in two groups (group A with ILM values lower than the median and group B with ILM values higher than the median). These two groups were characterized by a strong difference in lean body mass (p < 0.0001), but were not significantly different for fat mass, body weight and BMI.

#### Index of fat mass (IFM)

We defined an index in order to obtain two groups not differing for lean mass:

$$\begin{array}{l} \mathrm{IFM}=\mathrm{BW}/\left(\mathrm{LM}–\mathrm{FM}\right)\;\mathrm{and}\;\mathrm{therefore}\;\mathrm{IFM}\\ \;=\left(\mathrm{LM}+\mathrm{FM}\right)/\left(\mathrm{LM}–\mathrm{FM}\right) \end{array}$$

Since an increase in body weight is generally associated to an increase both in lean mass and in fat mass, but the extent of the increase is higher for fat mass, the higher is body weight, the higher is the numerator and the lower is the difference between lean mass and fat mass, and, subsequently, the higher is the value of IFM.

Thus, we calculated the value of IFM for every subject studied, we found its median and subsequently split our population into two groups (group C with IFM values lower than the median and group D with IFM values higher than the median). These two groups are characterized by a strong difference in fat mass, body weight and BMI (p < 0.0001), while there are no significant differences for the lean mass composition.

#### Statistical analysis

Data distribution was assessed using Shapiro-Wilk’s test. Data were reported as media and standard deviation (SD). Data with deviations from normality were shown as median and range. Differences between the two groups in all tables were calculated using independent Student’s t-test or Mann-Whitney’s U-test. A p value lower than 0.05 was considered significant for t-test or U-test. Holm’s test [24] was applied to adjust p values for multiple comparisons. Holm’s statistical procedures rejected an hypothesis only if its p-value was less than their corresponding critical values. Holm’s test was supported also by a discriminant analysis reported in Tables 1, 2 and 3 Pearson’s correlation coefficient (r) was reported for all parameters quantified. The data were statistically analysed using SPSS 20.0 (SPSS inc. Chicago, IL, USA).

**Table 1:** Discriminant function analysis for body composition of healthy women split into two groups based on the Index of Lean Mass (ILM)

**Parameter**	Standardized canonical discriminant function coefficient (*)
Index of Lean Mass (ILM)	0.81
Total lean mass, Kg	0.44
Height, cm	0.31
BMD upper limb, g/cm^2^	0.25

(*) Parameters with coefficient > 0.25. Lambda of Wilks (p < 0.001).

BMD: Bone Mineral Density.

**Table 2:** Discriminant function analysis for urinary lithogenic risk factors on a 24-hour urine collection in healthy women split into two groups based on the Index of Lean Mass (ILM)

**Parameter**	Standardized canonical discriminant function coefficient (*)
Potassium, mEq/24 h	0.52
Magnesium, mg/24 h	0.51
Phosphorus, mg/24 h	0.50
Creatinine, mg/24 h	0.48
Citrate, mg/24 h	0.44
Oxalate, mg/24 h	0.35
Sodium, mEq/24 h	0.26

(*) Parameters with coefficient > 0.25. Lambda of Wilks (p = 0.003).

**Table 3:** Discriminant function analysis for body composition in healthy women split into two groups based on the Index of Fat Mass (IFM)

**Parameter**	Standardized canonical discriminant function coefficient (*)
Total fat mass, Kg	0.67
BMI, Kg/m^2^	0.51
Weight, Kg	0.41
Total lower limb mass, Kg	0.41
Total trunk mass, Kg	0.37
Index of Fat Mass (IFM)	0.26

(*) Parameters with coefficient > 0.25. Lambda of Wilks (p < 0.001).

BMI: Body Mass Index.

## Results

The average age of the 78 women studied was 46 ± 6 years (range 31–59). 24% of them (19 women) had been menopausal since at least one year.

### ILM and IFM validation

Indexes validation was carrying out as follows. Weight, total lean mass and total fat mass are parameters strongly correlated to each other (weight *vs* total lean mass, r = 0.839 and p < 0.0001; weight vs total fat mass, r = 0.909 and p < 0.0001; total fat mass *vs* total lean mass, r = 0.538 and p < 0.0001, where r is a Pearson’s correlation coefficient). Multiple regression can be performed using least-squares method: total lean mass is dependent variable and weight is predictor, ILM (Index of Lean Mass) is reported in the model as covariate variable. This multiple regression is highly significant with p < 0.0001, R^2^ = 0.989 and R = 0.995. A simple linear regression with only weight as independent variable resulting R^2^ = 0.70 and R = 0.84. So ILM is very important in the explanation of the model. Total lean mass adjusted for weight correlates significantly with different parameters of urinary excretion and density bones. These Pearson’s correlations provide results equal to values obtained from the correlations of ILM with same parameters. ILM is independent from the weight and also from BMI. For example two subjects may have the same weight’s and height’s values (same BMI), but lean total mass completely different. ILM is a parameter more specific for total lean mass. Now it is clear that subjects with high lean total mass not have necessary low total fat mass. The second index IFM correlates highly significant with total fat mass, r = 0.689 and p < 0.0001 and it is not correlated with total lean mass. These correlations are confirmed by Discriminant Function Analysis by standardized canonical discriminant function coefficients reported in Tables 1, 2 and 3.

Table 4 shows the values of body composition and Table 5 shows the urinary lithogenic risk factors after partition of the women according to the median (1296) of the Index of Lean Mass. Groups A and B did not differ in body weight and BMI, but women in group B showed height and lean mass significantly higher (159 ± 6 vs 163 ± 5 cm and 40 ± 4 vs 45 ± 5 kg, p < 0.0001). Moreover, the group with high ILM showed a bone mineral density significantly higher in both upper and lower limbs and in ribs (Table 4).

**Table 4:** Body composition of healthy women split into two groups based on the Index of Lean Mass (ILM)

	**Group A**	**Group B**	p (*)	**p critical values (0.0029 < p < 0.0025)**
	**Low lean mass**	**High lean mass**		
	**ILM < 1296**	**ILM > 1296**		
	**N. 39**	**N. 39**		
ILM	1035 ± 205	1625 ± 313	**<0.0001**	###
Age, years	46 ± 6	46 ± 6	0.956	
Menopausal, n., % (°)	11 (28)	8 (21)	0.590**	
Years from menopause	1.4 ± 3	0.9 ± 2	0.416	
Weight, Kg	65 ± 10	68 ± 11	0.268	
Height, cm	159 ± 6	163 ± 5	**<0.0001**	###
BMI, Kg/m^2^	26 ± 4	25 ± 4	0.612	
Total trunk mass, Kg	31 ± 5	32 ± 6	0.289	
Total lower limb mass, Kg	22 ± 4	23 ± 4	0.460	
Total lean mass, Kg	40 ± 4	45 ± 5	**<0.0001**	###
Total fat mass, Kg	23 ± 6	21 ± 7	0.07	
BMD upper limbs, g/cm^2^	0.72 ± 0.04	0.76 ± 0.08	**0.003**	###
BMD ribs, g/cm^2^	0.64 ± 0.08	0.68 ± 0.07	**0.035**	
BMD lower limbs, g/cm^2^	1.10 ± 0.07	1.15 ± 0.1	**0.026**	
BMD pelvis, g/cm^2^	1.20 ± 0.15	1.24 ± 0.18	0.307	
BMD lumbar vertebrae, g/cm^2^	1 ± 0.13	1.04 ± 0.15	0.217	
BMD femur, g/cm^2^	0.91 ± 0.12	0.95 ± 0.11	0.232	
T-score lumbar vertebrae (+)	−0.17 (−2.50 – 2.31)	−0.45 (−2.54 – 2.87)	0.442***	
Z-score lumbar vertebrae (+)	0.41 (−1.92 – 2.73)	0.34 (−1.49 – 3.21)	0.407***	
T-score femur (+)	−0.16 (−1.92 – 2.07)	−0.09 (−1,50 – 1.52)	0.165***	
Z-score femur (+)	0.27 (−1.52 – 2.43)	0.37 (−1.27 – 1.93)	0.225***	

Data were reported as mean ± standard deviation (SD), unless otherwise specified.

°Data were reported as number of subjects (frequency).

+Data were reported as median and range.

*p value was calculated with nondependent Student’s t test, unless otherwise specified.

**χ^2^ test was applied to evaluate p value.

***Mann-Whithey’s u-test was applied to evaluate p value.

###Significant differences with p adjusted by Holm’s test.

BMD: Bone Mineral Density.

**Table 5:** Urinary lithogenic risk factors on a 24-hour urine collection in healthy women split into two groups based on the Index of Lean Mass (ILM)

	**Group A**	**Group B**	P (*)	**p critical values (0.0038 < p < 0.003)**
	**Low lean mass**	**High lean mass**		
	**ILM < 1296**	**ILM > 1296**		
	**N. 39**	**N. 39**		
Volume, ml	1603 ± 698	1640 ± 835	0.832	
Creatinine, mg/24 h	991 ± 194	1138 ± 191	**0.001**	**###**
Urea, mg/24 h	22 ± 6	23 ± 6	0.486	
Sodium, mEq/24 h	124 ± 49	143 ± 45	0.072	
Potassium, mEq/24 h	47 ± 13	60 ± 18	**<0.001**	**###**
Calcium, mg/24 h	156 ± 62	188 ± 91	0.076	
Phosphorus, mg/24 h	520 ± 174	665 ± 186	**<0.001**	**###**
Magnesium, mg/24 h	66 ± 20	85 ± 26	**<0.001**	**###**
Chloride, mEq/24 h	131 ± 50	151 ± 49	0.074	
Uric acid, mg/24 h	456 ± 136	508 ± 116	0.077	
Citrate, mg/24 h	620 ± 178	807 ± 323	**0.002**	**###**
Oxalate, mg/24 h	21 ± 7	27 ± 11	**0.015**	
Sulfate, mmol/24 h	16 ± 4	18 ± 5	0.085	
Ammonium, mmol/24 h	27 ± 11	30 ± 9	0.275	
pH 24 h	5.95 ± 0.51	5.96 ± 0.45	0.961	

Data were reported as mean ± standard deviation (SD).

*p value was calculated with independent Student’s t-test.

###Significant differences with p adjusted by Holm’s test.

The subjects of group B (with high lean mass) also showed urinary excretion of creatinine, potassium, phosphorus, magnesium, citrate and oxalate significantly higher than the ones of group A (Table 5).

The analysis of three-day dietary diaries did not show differences in the intake of water. Potential Renal Acid Load (PRAL calculated) [25], proteins, carbohydrates, lipids, sodium, potassium, calcium, phosphorus, and magnesium did not show differences between the two ILM groups (Table 6). The percentage of subjects that regularly performed physical exercise (according to WHO guidelines [26]) was not significantly different as well (Group A 31% vs Group B 49%, p = 0.105).

**Table 6:** Dietary intake of healthy women split into two groups based on the Index of Lean Mass (ILM)

	**Group A**	**Group B**	p (*)
	**Low lean mass**	**High lean mass**	
	**ILM < 1296**	**ILM > 1296**	
	**N. 39**	**N. 39**	
Water, ml	1470 ± 560	1470 ± 670	1.000
PRAL, mEq	12.75 ± 17.45	6.69 ± 18.85	0.145
Proteins, g/24 h	82 ± 22	89 ± 30	0.215
Carbohydrates, g/24 h	258 ± 89	272 ± 92	0.493
Lipids, mEq/24 h	82 ± 29	93 ± 29	0.101
Sodium, mEq/24 h	80 ± 44	95 ± 61	0.212
Potassium, mEq/24 h	66 ± 18	73 ± 23	0.150
Calcium, mg/24 h	757 ± 416	842 ± 347	0-330
Phosphorus, mg/24 h	1150 ± 418	1105 ± 350	0.610
Magnesium, mg/24 h	261 ± 97	295 ± 126	0.181

Data were reported as mean ± standard deviation (SD).

*p value was calculated with independent Student’s t-test.

PRAL: Potential Renal Acid Load. The Dietosystem software (DS Medica) calculates PRAL applying the original model described by Remer and Manz (ref).

Table 7 shows the values of body composition and Table 8 the urinary lithogenic risk factors after subdivision of the women according to the median (3.28) of the Index of Fat Mass. The two groups did not differ in height, but the group with a higher IFM showed significantly greater values of BMI, total trunk mass, total leg mass and total body fat mass. The bone mineral density of the pelvis, lumbar vertebrae and femur, and the respective T and Z score, were significantly better in Group D, the one with high IFM (Table 7).

**Table 7:** Body composition in healthy women split into two groups based on the Index of Fat Mass (IFM)

	**Group C**	**Group D**	p (*)	**p critical value (0.0036 < p < 0.0025)**
	**Low fat mass**	**High fat mass**		
	**IFM < 3.28**	**IFM > 3.28**		
	**N. 39**	**N. 39**		
IFM	2.50 ± 0.42	5.14 ± 4.03	**<0.0001**	**###**
Age, years	45 ± 6	47 ± 6	0.23	
Menopause, n., % (°)	8 (21)	11 (28)	0.429**	
Years from menopause	0.8 ± 3	1.5 ± 3	0.336	
Weight, Kg	60 ± 7	73 ± 10	**<0.0001**	**###**
Height, cm	161 ± 6	161 ± 6	0.467	
BMI, Kg/m^2^	23 ± 2	28 ± 3	**<0.0001**	**###**
Total trunk mass, Kg	28 ± 4	35 ± 6	**<0.0001**	**###**
Total lower limb mass, Kg	21 ± 3	25 ± 3	**<0.0001**	**###**
Total lean mass, Kg	41 ± 5	43 ± 5	0.064	
Total fat mass, Kg	17 ± 3	27 ± 5	**<0.0001**	**###**
BMD upper limbs, g/cm^2^	0.74 ± 0.08	0.73 ± 0.06	0.347	
BMD ribs, g/cm^2^	0.66 ± 0.08	0.66 ± 0.07	0.886	
BMD lower limbs, g/cm^2^	1.13 ± 0.09	1.13 ± 0.09	0.896	
BMD pelvis, g/cm^2^	1.18 ± 0.15	1.27 ± 0.17	**0.013**	
BMD lumbar vertebrae, g/cm^2^	0.98 ± 0.13	1.06 ± 0.14	**0.018**	
BMD femur, g/cm^2^	0.90 ± 0.10	0.96 ± 0.12	**0.025**	
T-score lumbar vertebrae (+)	−0.55 (−2.54 – 2.87)	0.25 (−2.50 – 2.68)	0.013***	
Z-score lumbar vertebrae (+)	−0.13 (−1.92 – 3.19)	0.63 (−1.46 – 3.21)	0.004***	
T-score femur (+)	−0.52 (−1.55 – 1.25)	0.24 (−1.92 – 2–07)	0.034***	
Z-score femur (+)	−0.18 (−1.27 – 1.93)	0.61 (−1.52 – 2.43)	0.014***	

Data were reported as mean ± standard deviation (SD), unless otherwise specified.

°Data were reported as number of patients (frequency).

+ Data were reported as median and range.

*p value was calculated with independent Student’s t-test, unless otherwise specified.

**χ^2^ test was applied to evaluate p value.

***Mann-Whitney’s u-test was applied to evaluate p-value.

###Significant differences with p adjusted by Holm’s test.

BMD: Bone Mineral Density.

**Table 8:** Urinary lithogenic risk factors on a 24-hour urine collection in healthy women, split into two groups based on the Index of Fat Mass (IFM)

	**Group C**	**Group D**	p (*)
	**Low fat mass**	**High fat mass**	
	**IFM < 3.28**	**IFM > 3.28**	
	**N. 39**	**N. 39**	
Volume, ml	1692 ± 793	1551 ± 740	0.418
Creatinine, mg/24 h	1034 ± 201	1095 ± 207	0.188
Urea, mg/24 h	22 ± 6	22 ± 6	0.807
Sodium, mEq/24 h	130 ± 45	137 ± 51	0.501
Potassium, mEq/24 h	55 ± 18	53 ± 17	0.677
Calcium, mg/24 h	178 ± 90	167 ± 67	0.537
Phosphorus, mg/24 h	574 ± 196	611 ± 192	0.402
Magnesium, mg/24 h	80 ± 28	71 ± 21	0.138
Chloride, mEq/24 h	138 ± 48	144 ± 53	0.622
Uric acid, mg/24 h	469 ± 120	495 ± 136	0.363
Citrate, mg/24 h	736 ± 292	691 ± 261	0.478
Oxalate, mg/24 h	23 ± 7	26 ± 12	0.162
Sulfate, mmol/24 h	17 ± 4	17 ± 5	0.526
Ammonium, mmol/24 h	28 ± 9	29 ± 12	0.881
pH 24 h	6.00 ± 0.43	5.92 ± 0.53	0.451

Data were reported as mean ± standard deviation (SD).

*p value was calculated with independent Student’s t-test.

The urinary lithogenic risk factors (Table 8) showed no differences between Group C (subjects with low IFM) and Group D (subjects with high IFM); besides, even dietary intakes did not reveal significant differences (Table 9). The percentage of subjects regularly performing physical activity appeared instead significantly higher in women with a low Index of Fat Mass (Group C 54% vs Group D 26%, p = 0.01).

**Table 9:** Dietary intake of healthy women split into two groups based on of the Index of Fat Mass (IFM)

	**Group C**	**Group D**	p (*)
	**Low fat mass**	**High fat mass**	
	**IFM < 3.28**	**IFM > 3.28**	
	**N. 39**	**N. 39**	
Water, ml	1530 ± 650	1410 ± 580	0.371
PRAL, mEq	11.30 ± 16.60	6.10 ± 20.02	0.217
Proteins, g/24 h	90 ± 29	81 ± 20	0.131
Carbohydrates, g/24 h	278 ± 92	252 ± 87	0.204
Lipids, mEq/24 h	93 ± 32	82 ± 25	0.112
Sodium, mEq/24 h	92 ± 59	82 ± 25	0.112
Potassium, mEq/24 h	71 ± 23	68 ± 19	0.521
Calcium, mg/24 h	880 ± 403	719 ± 349	0.062
Phosphorus, mg/24 h	1199 ± 440	1056 ± 308	0.100
Magnesium, mg/24 h	288 ± 114	268 ± 112	0.426

Data were reported as mean ± standard deviation.

*p value was calculated with independent Student’s t-test.

Discriminant function analysis for body composition and urine chemistries regarding ILM and IFM are reported in Tables 1 and 2 for ILM and Table 3 for IFM.

## Discussion

Our data demonstrate that the urine composition in our cohort of female healthy volunteers is significantly influenced by the body composition in lean mass. A high lean mass promotes a high excretion both of some lithogenesis promoters, such as phosphate and oxalate, and of some lithogenesis inhibitors, such as magnesium, potassium and citrate. A positive trend also seems to occur with other urinary analytes such as sodium, chloride, uric acid and sulphate, although at the limit of statistical significance, perhaps because of the relatively low number of subjects studied.

It seems plausible to argue that these findings were not due to differences in dietary intake, as demonstrated by a nutritional analysis performed through a 3-day dietary diary. We can therefore assume that lean mass plays an active role in determining urine composition, while fat mass seems to act as a metabolically inactive bystander.

This hypothesis partially conflicts with the current paradigm that considers nephrolithiasis as a systemic disorder strongly linked to metabolic syndrome. There are data showing that a high insulin resistance, possible expression of a high fat mass, leads to lower urinary pH, to a high acid load and ammonium excretion [27]. This would expose subjects with a high fat mass to a higher risk of uric acid stones, although there are also data linking various features of the metabolic syndrome to calcium nephrolithiasis too [28]. These findings may explain the strong epidemiologic correlation between obesity and kidney stones [3-5].

On the other hand, there are also some reports indirectly suggesting that fat mass does not affect lithogenic risk until BMI rises to the range of morbid obesity. For example, Taylor et al. found that lithogenic risk does not rise for a body weight up to 67.7 kg and a BMI up to 27.7 kg/m^2^. Moreover, some recent data show that obesity does seem to determine a higher risk of nephrolithiasis in a children cohort, but surprisingly does not influence urine chemistries at all [22]. Finally, another recent report shows that in obese stone formers body composition does not influence stone chemistry until very high levels of BMI (> 40 kg/m^2^) are reached [29], thus indirectly supporting our finding that urine chemistry is poorly influenced by fat mass. In fact, it is remarkable to point out that in our research there was an average difference in body weight of about 13 kg between group C (low fat mass) and group D (high fat mass) (Table 7).

The relationship between lean mass and urine composition has been indeed poorly investigated in literature. However, our findings partially match with those by Lemann jr et al., who demonstrated that oxalate and calcium excretion in males is directly related to creatinine excretion, an index of lean mass composition, in a cohort of healthy subjects [8]. Thus, the increase of lean mass might cause a rise in the risk for calcium nephrolithiasis.

We may speculate that a high lean mass leads to higher protein catabolism, thus influencing the differences in urine composition we found in our research. We must also point out that subjects in group B, the ones with a high lean mass, had also a higher prevalence of physical exercise, although not statistically significant. It is plausible that physical exercise may influence a more active muscular metabolism, thus causing a higher excretion of metabolites such as oxalate, phosphate and citrate.

The analysis of bone mineral density in our subjects confirmed the assumption, already well established in literature [18], that the higher is the body mass, the better is the quality of the bone, particularly in the spine (Table 7). The women with high IFM had a significantly higher bone mineral density in lumbar vertebrae, pelvis and femur. This group also shows a poor percentage of subjects regularly performing physical activity (26% vs 54%). This tallies with published data showing that in premenopausal sedentary women bone mineral density correlates with fat mass [30]. However, we have to consider that in our model total body weigh increase when fat mass rise suggesting a non-linear dose–response relationship of fat mass on BMD as previous suggested [17]. On the other hand, the group with high lean mass shows better mineral density in upper and lower limbs and ribs. This group also includes subjects taller than the ones with low lean mass. It has already been demonstrated that height correlates with lean mass and mineral density of extra-axial bones [30,31]. We can also suppose that a better bone mineral density in limbs and ribs is, at least partially, due to physical exercise with a subsequent increase in muscle mass and mechanic anabolic stimulus on the bone [32,33].

We are aware of some limits that are implied in our study. First, the number of subjects studied is rather low. Secondly, the groups were split on the basis of mathematical indexes built to highlight lean mass and fat mass and not on the basis of direct measures. Moreover, we did not carry out an analysis distinguishing pre-menopausal and post-menopausal women. Finally, the analysis of a three-day dietary diary may not exhaustively capture the real dietary habits of a subject; nevertheless the diaries were interpreted by a dietitian during a meeting and the results do not change even after correction for body weight.

## Conclusions

This paper suggests that in healthy women with a similar dietary intake, fat mass does not seem to influence the urinary excretion of lithogenic risk factors, which on the other hand seems to be much more dependent on the level of lean mass. Moreover, bone mineral density seems to be influenced by fat mass, while lean mass might play a positive role particularly on the extra-axial skeleton, as a possible result of the muscular activity. However, the field of interactions between body composition and mineral metabolism is far from being fully understood. Further research on larger cohorts both of healthy subjects and kidney stone formers or people with osteoporosis will clarify the specific role of lean mass and fat mass.

## Abbreviations

BMD: Bone mineral density; DEXA: Dual Energy X-ray Absorptiometry; ILM: Index of lean mass; IFM: Index of fat mass; BMI: Body mass index; BW: Body weight; LM: Lean mass; FM: Fat mass; PRAL: Potential renal acid load.

## Competing interests

The authors declare they have not competing interests.

## Authors’ contributions

AN, ATi, AG carried out study design, data interpretation and manuscript preparation; FA, ED, ATe: participants enrollment and clinical assessment; SP and GL: laboratory tests and supervision; MP: DEXA execution and interpretation; GF and PB: statistical analysis; LB and LS: critical revision; TM: critical revision, organization and supervision. All authors read and approved the final manuscript.
